# Genomic characterization of *Orthonairovirus haemorrhagiae* (Crimean-Congo hemorrhagic fever virus) outbreak in North Macedonia

**DOI:** 10.1128/mra.00749-24

**Published:** 2024-11-20

**Authors:** Golubinka Boshevska, Petra Emmerich, Ronald von Possel, Elizabeta Jancheska, Teodora Buzharova, Dragan Kochinski, Gábor Endre Tóth, Dániel Cadar, Dugagjin Osmani

**Affiliations:** 1 Institute of Public Health, Skopje, North Macedonia; 2 Faculty of Medical Sciences, Goce Delchev University, Shtip, North Macedonia; 3 Bernhard Nocht Institute for Tropical Medicine, WHO Collaborating Centre for Arbovirus and Hemorrhagic Fever Reference and Research, Hamburg, Germany; 4 Department of Tropical Medicine and Infectious Diseases, Center of Internal Medicine II, University of Rostock, Rostock, Germany; DOE Joint Genome Institute, Berkeley, California, USA

**Keywords:** metagenomics, North Macedonia, genomic surveillance, Crimean-Congo hemorrhagic fever virus, evolution, outbreak

## Abstract

Crimean-Congo hemorrhagic fever virus (CCHFV) is a significant tick-borne virus causing severe hemorrhagic disease with high fatality rate. This report presents the genomic characterization of CCHFV strain from North Macedonia’s first outbreak in over 50 years, emphasizing the importance of genomic surveillance in tracking virus evolution and spread patterns in this region.

## ANNOUNCEMENT

Crimean-Congo hemorrhagic fever virus (CCHFV) classified in the *Orthonairovirus haemorrhagiae* species (genus *Orthonairovirus*, family *Nairoviridae*) is a tick-borne virus, known to cause severe hemorrhagic fever in humans with high mortality rates (1). The virus is widely distributed by ticks of the *Hyalomma* genus as the principal vector and reservoir of CCHFV across Africa and Asia, with sporadic outbreaks in south and southeastern Europe (2–6). The current climatic conditions in the West Balkan countries make it a hotspot for vector-borne diseases, showing a concerning increase in CCHFV sporadic cases and outbreaks, highlighting the need for enhanced surveillance and preventive measures.

**Fig 1:**
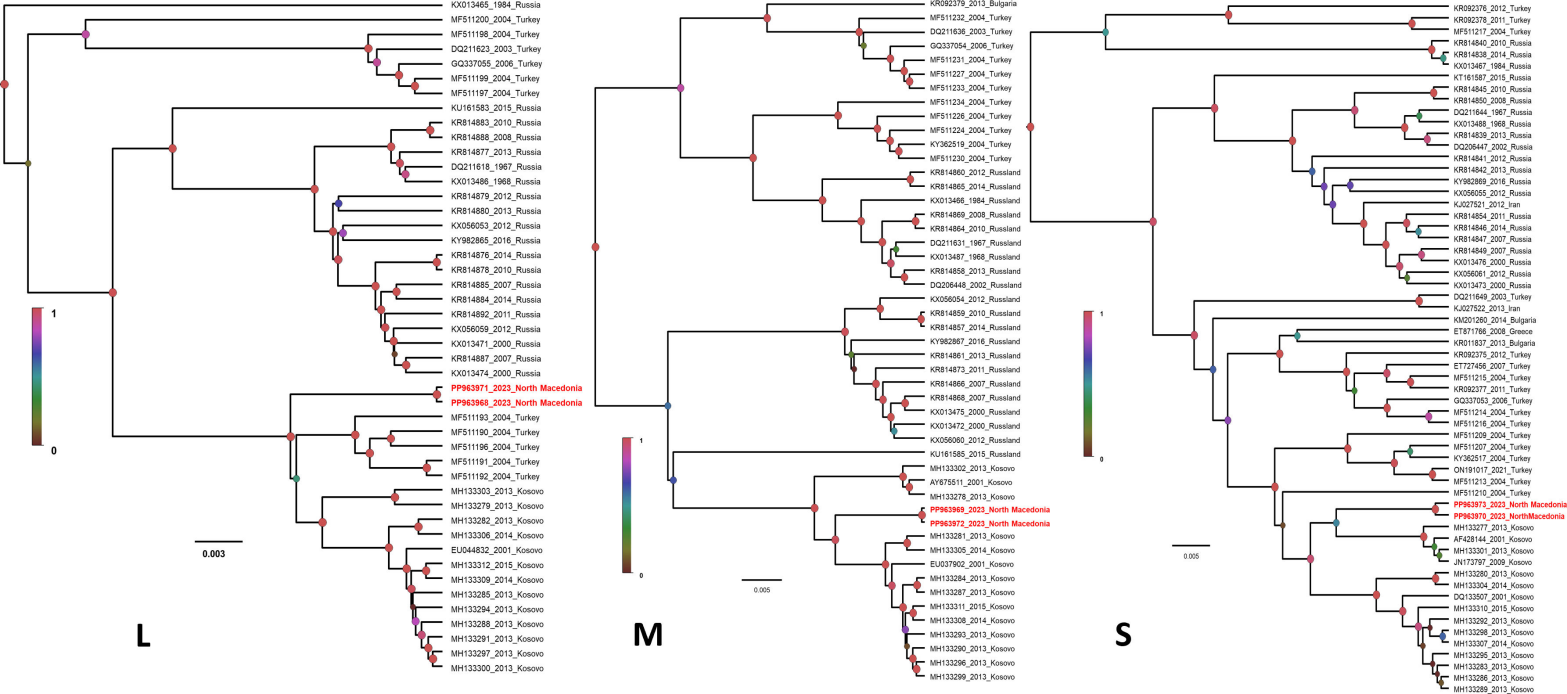
Phylogenetic analysis of the CCHFV genotype V (Europe 1) based on S, M, and L segments, including the genomes of human CCHF cases from North Macedonia. Nucleotide sequences were aligned using MAFFT in Geneious Prime v.2024.03. Phylogenetic analyses were conducted with the best-fit nucleotide substitution models, selected based on their lowest Akaike information criterion and Bayesian information criterion scores using jModelTest v.2.1.10 and ProtTest v.3.4.2. Nucleotide phylogenies were constructed using Bayesian inference with the Markov chain Monte Carlo method, implemented in Beast v.1.10.3. Figtree v.1.4.3 was used to visualize the tree output files. Each phylogeny is labeled with the corresponding viral segments L, M, and S, respectively. Statistical support of grouping from Bayesian posterior probabilities is indicated at the nodes and in the color legend. Taxon information includes GenBank accession number, year of detection, and country of origin. The human CCHFV sequences from North Macedonia generated during this study are in bold (red marked). The scale bar indicates the mean number of nucleotide substitutions per site.

In July 2023, a CCHFV-positive sample was collected from a patient originating from Kuchica village, near the city of Shtip, North Macedonia. A healthcare worker was exposed and contracted the infection 5 days after index case acceptance to the Clinic for Infectious Diseases in Skopje (7). In both cases, RNA from sera samples was extracted using QIAamp Viral RNA Mini Kit and subjected to high-throughput sequencing using the Illumina sequencing platform in the NGS Core Facility of BNITM, on 27 January 2024. After random amplification of the RNA, the obtained cDNA was used for library preparation using the QIAseq FX DNA Library Kit as described elsewhere (8). Metagenomic sequencing (9) was performed on a NextSeq 2000 sequencing platform using the 200-cycle NextSeq 2000 P2 reagent kit. Raw data underwent quality control to remove low-quality reads and polyclonal sequences using CLC Genomics Workbench 22. Paired-end reads were merged, and contigs (≥400 nucleotides) obtained with Megahit v.1.2.9 (10) were classified using Diamond’s long-read algorithm against the National Center for Biotechnology Information’s non-redundant (NR) protein database v.2024.01. MEGAN6 Community Edition (11) facilitated binning, visualization, and downstream analysis. Sequence analysis and genome assembly of CCHFV were performed with Geneious Prime v.2024.03. Phylogenetic analyses were conducted using the Bayesian Markov chain Monte Carlo tree-sampling method with BEAST v.1.10. The GTR + G + I model was determined to be the best fit for sequence evolution. We successfully sequenced and assembled the complete genomes of index patients while the viral genome recovery from the health worker was about 80% (Table 1). The CCHFV strain showed high nucleotide similarity of 98.2%–99.50% with its closest related sequences from Turkey and Kosovo, respectively. Bayesian phylogenetic analyses showed that the Macedonian CCHF-derived strains, together with Kosovar CCHFV variants, grouped into a distinct monophyletic clade within CCHFV genotype V (Europe 1) phylogeny (Fig. 1), suggesting a common origin and potential cross-border transmission routes. Furthermore, the Macedonian strains form a distinct well-supported subclade. The phylogeny suggests the relative long-term circulation of CCHFV in the region and the Macedonian variant as a reassortant descendant of an ancestor that probably emerged in Turkey and Kosovo. The genomic data provide critical insights into the epidemiology and evolution of CCHFV in Europe, particularly in West Balkan countries. The genetic diversity observed among the West Balkan countries underscores the need for continuous genomic surveillance to monitor viral evolution and inform control strategies. Our findings highlight the importance of regional collaboration in addressing tick-borne diseases, given the transnational nature of CCHFV transmission. The data generated will aid in the development of targeted interventions and contribute to the global efforts to control the spread of CCHFV.

**TABLE 1:** Summary of genomic sequence information of CCHFV from the index patient and healthcare worker from north Macedonia

Sequence Read Archive acc. no.	GenBank acc. no.	Sample	Total reads	Total reads trimmed	Virus reads	Segment	Average coverage depth	Completeness (%)	GC content (%)	Total length (bp)	Closest related CCHFV
SRX25242422	PP963968	Index patient	2,022,154	1,937,510	45,407	S	402	100	47.2	1,814	MH133301
	PP963969	Index patient				M	515	100	48.3	5,388	EU037902
	PP963970	Index patient				L	268	100	43.1	12347	MH133294
SRX25242423	PP963971	Healthcare worker	2,417,186	2,308,622	597	S	1.4	68.9	47.6	1,244	MH1,33301
	PP963972	Healthcare worker				M	3.1	85.6	46.5	4,689	EU037902
	PP963973	Healthcare worker				L	2.4	78.4	42.8	8,998	MH133294

## Data Availability

Raw sequence data are available under National Center for Biotechnology Information Sequence Read Archive number PRJNA1131364 (SRX25242422 index case, SRX25242423 healthcare worker) and the consensus sequences of CCHFV genomes under GenBank accession numbers for index case PP963968-PP963970 and PP963971-PP963973 for the healthcare worker, respectively.
