# Correction for Boshevska et al., “Genomic characterization of *Orthonairovirus haemorrhagiae* (Crimean-Congo haemorrhagic fever virus) outbreak in North Macedonia”

**DOI:** 10.1128/mra.00486-25

**Published:** 2025-06-18

**Authors:** Golubinka Boshevska, Petra Emmerich, Ronald von Possel, Elizabeta Jancheska, Teodora Buzharova, Dragan Kochinski, Gábor Endre Tóth, Dániel Cadar, Dugagjin Osmani

## AUTHOR CORRECTION

Volume 13, no. 12, e00749-24, 2024, https://doi.org/10.1128/mra.00749-24. Page 1, paragraph 2, sentence 2: “(7)” should read “(7, 12).”

Page 5: The following reference was inadvertently omitted.

12. Jakimovski D, Banović P, Spasovska K, Rangelov G, Cvetanovska M, Cana F, Simin V, Bogdan I, Mijatović D, Cvetkovikj A, Djadjovski I, Christova I, Meletis E, Kostoulas P, Zana B, Lanszki Z, Görföl T, Tauber Z, Kemenesi G. 2024. Detection of autochthonous virus strain responsible for the recent outbreak of Crimean-Congo haemorrhagic fever in North Macedonia, July to August 2023. Research Square https://doi.org/10.21203/rs.3.rs-4360716/v1.

